# Role of fast-spiking interneurons in modulating across-trial variability and within-trial correlations in the striatum

**DOI:** 10.1371/journal.pcbi.1014099

**Published:** 2026-03-27

**Authors:** Lihao Guo, Arvind Kumar

**Affiliations:** 1 Division of Computational Science and Technology, School of Electrical Engineering and Computer Science, Digital Futures, KTH Royal Institute of Technology, Stockholm, Sweden; 2 Science for Life Laboratory, Stockholm, Sweden; 3 Current affiliation: Okinawa Institute of Science and Technology, Okinawa, Japan; 4 Digital Futures, KTH Royal Institute of Technology, Stockholm, Sweden; Research Center Jülich, GERMANY

## Abstract

The striatum comprises a network characterized by a highly shared feedforward inhibition (FFI) mediated by fast-spiking interneurons (FSI), which constitute only 1% of the striatal population. We investigated the dynamical consequences of this extensively shared FFI beyond inducing synchrony in a local striatal microcircuit. Our findings reveal that increased FFI sharing enhances the across-trial variability of striatal responses, activity of medium spiny neurons (MSNs), to cortical inputs, and endows the striatal network with the capacity to modulate output correlations in a bidirectional manner. Specifically, weakly shared cortical inputs become more correlated, whereas strongly shared cortical inputs are decorrelated in the presence of FSIs. These dynamic modulatory effects on MSNs, in turn, substantially alter the spiking statistics of downstream neurons in the globus pallidus, regarding across-trial variability and burstiness.

## Author summary

The striatum is the main input of the basal ganglia and plays a crucial role in decision-making and motor learning. The striatum is primarily a network of mutually inhibiting medium spiny neurons (MSNs). About 5% neurons are inhibitory interneurons, and among those, 1% neurons are called Fast Spiking Interneurons, which provide feedforward inhibition: they are activated by cortical inputs and quickly suppress MSNs. A key feature of the striatum is that feedforward inhibition is highly shared by MSNs. We show two important consequences of this connectivity for information processing in the striatum. First, shared inhibition from FSIs increases trial-to-trial variability in the population response of MSNs. This means the striatum can produce more diverse responses to the same stimulus. Second, FSIs have a dual effect on correlations between MSNs: when cortical inputs are weakly shared, FSIs increase correlations, but when inputs are strongly shared, FSIs reduce correlations. Thus, FSIs can either synchronize or desynchronize activity depending on the input context. These two effects propagate downstream and make the activity of the globus pallidus more variable and burst-like. Our results suggest that FSIs may support exploration and flexibility, especially during early learning when input patterns are unstable.

## Introduction

The striatum, the primary input station of the basal ganglia, is a mainly inhibitory network of medium spiny neurons (MSN) and several types of interneurons. The MSNs that express D1 or D2-type dopamine receptors form the majority of the striatal network (≈95%), and interneurons such as cholinergic interneurons (ChIN), fast-spiking interneurons (FSI), and low-threshold spiking neurons (LTS) make up the remaining 5% [1].

The FSIs form the classical feedforward inhibitory (FFI) motif in the striatum. That is, cortico-striatal inputs project onto both the MSNs and FSIs, which in turn inhibit MSNs with forward projections. Thus, MSNs receive both feedforward excitation from the cortex as well as feedforward inhibition from FSIs. Such a motif of feedforward inhibition is ubiquitous in the brain [2], playing a critical role in defining the temporal window for spike discharge, modulating circuit gain [2], and selectively gating neural activity patterns [3]. Moreover, when synapses exhibit short-term facilitation/depression, FFI can modulate the dynamic range of neural output [4,5] and improve the detection of signals with a low signal-to-noise ratio [6].

What distinguishes the striatal FFI from other brain networks is that only 1% of the striatal neurons are FSIs, yet the projection covers all MSNs. FSIs project onto MSNs with a connection probability greater than 60% within their axonal reach [7–9]. Such a connection structure is starkly different from the neocortex, cerebellum, and hippocampus, where the FSI→pyramidal neuron (PYD) connection probability is between 10–20%. Besides the dense projections, the connection strength of FSI inputs is five to tenfold stronger than the recurrent inhibition among MSNs [10]. Together with the high firing rate of FSIs, this sparse population elicits a potent inhibition over MSNs comparable to the lateral inhibition between MSNs.

Even though the levels of the two inhibitions are comparable, the connectivity patterns of MSNs and FSIs exhibit opposite features. Lateral connections among the MSNs are weak and sparse. By contrast, FSI synapses are strong and dense. Previous studies have, therefore, suggested that FSIs promote correlated activity while MSNs drive decorrelation [11,12]. It originates from a straightforward intuition: the level of correlation in a network relies on the input sharing [13], which is significant for FSI inhibition but not for MSN inhibition. However, it remains unclear how stimulus-evoked shared inputs from the cortex are affected by the shared feedforward inhibition.

We found two dynamical consequences of the striatal FFI architecture for evoked activities. First, the highly shared nature of FFI projections increases the across-trial variability of MSNs’ responses at the population scale, regardless of the sharing of cortical inputs. Second, the correlating effect of FSIs competes with the decorrelating effect of MSNs such that weakly shared inputs get more correlated and strongly shared inputs get decorrelated compared to the no FSI case. Thus, FFI sharing results in a bidirectional modulation of correlation in the striatum. These dynamical effects are seen in the statistics of downstream readout neurons in the globus pallidus: shared FFI projections make the globus pallidus neurons both more variable and bursty.

Based on these observations, we speculate that FSIs may play a bigger role in the early phase of learning, when correlation structure from cortical inputs is less stable (weakly correlated), and high across-trial variability could be used for exploration. As these findings solely depend on the connectivity structure of FSIs, the implications of our results extend beyond the striatum and apply to other rare cell types with similar connectivity (i.e., those that comprise less than 5% of the total population).

## Results

**Fig 1:**
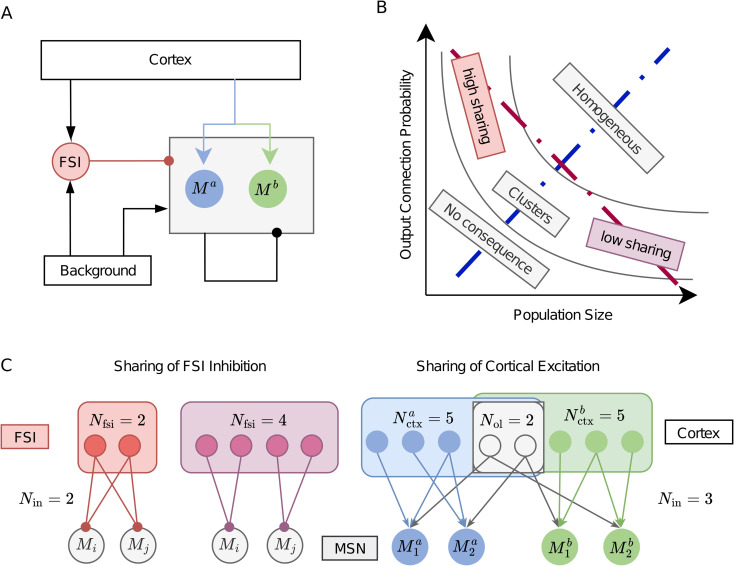
Simplified cortico-striatal circuit. **(A)** The diagram of a simplified cortico-striatal circuit. Each arrow denotes the connection from one population to another, where the arrowhead is excitatory, and the circlehead is inhibitory. In the circuit, MSNs receive direct excitatory inputs from the cortex and relayed inhibitory inputs via FSIs. *M*^a^ and *M*^b^ denote two MSN groups differentiated by their cortical sources as in **C** Right. Background inputs maintain the spontaneous activities of the striatum. The inhibitory projections from FSIs to MSNs are much stronger than the recurrent connections between MSNs, denoted by the arrow widths. **(B)** The type of connectivity that may emerge is a function of the relative size of a neuron population (x-axis) and its probability to connect (y-axis) to other neurons in the network. **(C)** Illustration of the feedforward connections from FSIs or cortical neurons to MSNs. (**C**:Left) Sharing of FSI inhibition, where the colored nodes denote FSIs, and gray nodes denote MSNs. The color of FSI to MSN projections corresponds to the high sharing (red) and low sharing (purple) scenarios shown in **B**, assuming indegree is fixed. (**C** Right) Sharing of feedforward excitation. Each MSN received a fixed number of cortical inputs. The presynaptic pools of the two MSN populations (blue and green) partially overlap. *N*_ctx_ denotes the size of the private pool, and *N*_ol_ denotes the shared pool of presynaptic cortical neurons for the two sources.

How a neuron population may affect the network structure and dynamics depends on its relative size in the network and how strongly or densely it projects to other neuron populations (Fig 1B). A large neuron population with a high outgoing connection probability would result in a network in which neurons will have a high overlap in their presynaptic inputs and postsynaptic targets. In this sense, at the structural level, the network would be considered homogeneous, i.e., neurons would not form clear clusters based on their inputs or outputs. Unless neurons have very different properties, activity in such a network would also be homogeneous, i.e., the activity of any two randomly sampled neurons would be statistically identical, precluding any activity-based clustering. On the other hand, a small population with a low connection probability would have a negligible effect on the network structure and dynamics, due to very few connections. For a medium-sized population and a moderate connection probability, these neurons would form clusters. That is, in the space spanned by population size and outgoing connection probability along the diagonal (Fig 1B, blue dashed line), we move from no connectivity to clusters to homogeneous connectivity. In the same space along the off-diagonal (Fig 1B, red dashed line), we move from high sharing to low sharing of connectivity. That is, a large population with low connection probability would form sparse connectivity with weak sharing of postsynaptic targets; by contrast, rare neuron types (e.g., < 5% of the whole network) with high connection probability would introduce highly shared connectivity. FSIs in the striatum fall in the later category – small population size but high connection probability.

Here, we characterise how the sharing of FFIs may affect the response of the MSNs when they receive shared inputs from thalamocortical projections. To this end, we simulated a simplified cortical-striatal circuit (Fig 1A) with 2500 MSNs and a variable number of FSIs. Neurons were connected randomly with conductance-based synapses, and synaptic weights were drawn from a lognormal distribution (see Synapse model). The neurons were driven by Poisson-type spiking input to simulate background activity (see Spontaneous activity), while stimulus-induced activity was modelled as convergent Poisson processes (see Input model). We systematically varied the number of FSIs while keeping the indegree of connections and neuron properties fixed.

### Across-trial variability

**Fig 2:**
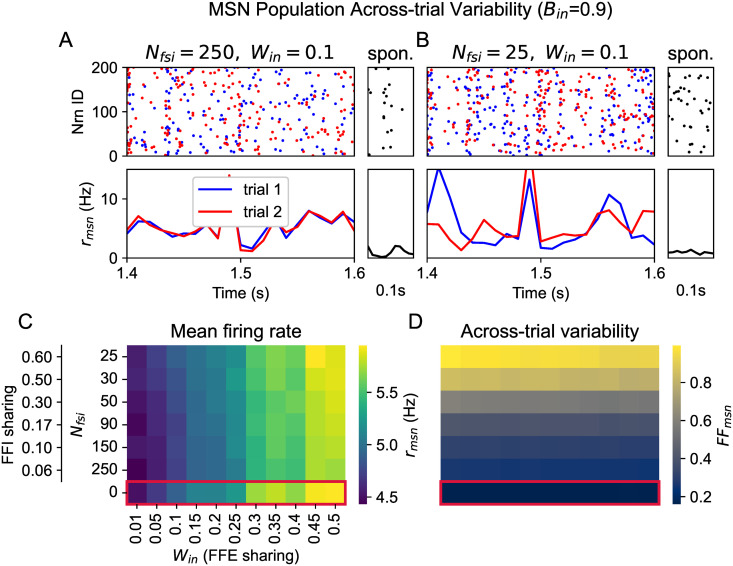
Across-trial variability of MSN activities during evoked states. **(A)** Top: raster plot (2 different trials denoted by red and blue) of MSN activities with $N_{\text{fsi}}=250,W_{\text{in}}=0.1,B_{\text{in}}=0.9$. Bottom: population rate of MSNs over time. The small panel shows spontaneous activity without any cortical input for 0.1s. **(B)** Same as **A** but for *N*_fsi_ = 25. **(C)** The color denotes the average population firing rates of MSN across trials given different FFE sharing (*W*_in_) and FFI sharing, i.e., the number of FSIs (*N*_fsi_). **(D)** Same as **C**, but for the across-trial variability of population rate trajectories.

To ask whether shared FFI can affect across-trial variability, we tuned the striatal network in a baseline state (see Spontaneous activity) with weak correlations and low firing rate [14], and injected an additional input that mimicked the activity evoked by cortical axons (Fig 1A). The cortical signal was fixed across trials, whereas the background noise had different realizations. The average firing rate of MSN during evoked activity was tuned to around 5 Hz, and MSNs could briefly spike up to 15 Hz (Fig 2A and 2B, bottom row), which is consistent with the bursting behavior of MSNs [15].

Next, we varied the population size of the cortical source and FSI (*N*_fsi_) while keeping the number of cortical or FSI inputs to each MSN fixed (see Input model). Thus, we systematically changed the sharing of feedforward excitation (FFE) and inhibition, while maintaining the average input constant. It ensured that the average MSN firing rate did not change significantly for different *N*_fsi_ given the same inputs (Fig 2C). For small *N*_fsi_, due to the sharing of FSI projections, MSNs became more correlated (see Bidirectional modulation of correlation). However, given the sparse connectivity (10% connection probability), MSN correlations did not affect the MSNs’ firing rate (see S3D Fig). For the control case without FSI (*N*_fsi_ = 0), we reduced the background input to maintain a response rate comparable to other cases when *N*_fsi_ >0. As expected, MSN firing rate increased with *W*_in_ due to multapses (see Input model).

By measuring the evoked responses of one MSN population across trials, we found that the variability (see Across-trial variability for the definition) increased with stronger sharing of FFI, as shown in Fig 2D. This effect is nontrivial as the sharing of cortical input mainly influenced the firing rate, and was not related to the across-trial variability of population rate (compare Fig 2C and 2D). As the only variable we have changed is *N*_fsi_, the increased variability is a direct consequence of shared FSIs. In this model, the higher the sharing of FFI, the larger the ongoing activity fluctuations at the population level, and therefore the larger the across-trial variability.

This population variability differs from the spike count variability of individual neurons as measured in [15]. For individual neurons, the determining factor is the signal-to-noise ratio (SNR), i.e., the ratio of stimulus-related input and background input [11]. In our case, the SNR remained constant as the input rate and indegree were constant. At the population scale, covariances among neurons play a critical role in the dynamics [16]. In short, the noisy activity of FSIs [17] was shared by MSNs in the form of population fluctuations across trials.

In the above simulations, all the neurons of a given type were homogeneous and only the synaptic weights/delays were heterogeneous (see Connectivity model). We relaxed this assumption and introduced heterogeneity in neurons (see Neuron model and S1 Fig). Neuronal heterogeneity reduced the variability by a small amount; however, qualitatively the results remained unchanged S2 Fig.

### Bidirectional modulation of correlation

It is well known that shared inputs (excitatory and inhibitory) result in correlated activity between neurons [13]. To avoid excessive correlation, excitation and inhibition must be balanced to cancel shared inputs and by suppressing the shared fluctuations [18,19]. In the striatum, shared FFI can induce synchrony, which recurrent inhibition among MSNs can counter [11]. However, it is unclear how FSIs and MSNs interact when the cortical inputs are shared between striatal neurons.

To address this question, we considered two groups of MSNs stimulated by cortical inputs (Fig 1A). In this simplified circuit, we did not make any assumption about the separation of D1- and D2-MSN by neuronal property or recurrent connectivity. However, the circuit is sufficient to understand the role that FSIs may play in modulating correlation. The inputs to the two MSN groups were parameterized by the mean rate (fixed), the sharing of input within (*W*_in_) and between (*B*_in_) the two MSN groups (see Fig 1C). As in the previous section, we systematically varied the sharing of cortical input and FSI inhibition by changing the population size of cortical sources or FSIs while maintaining the corresponding indegree of projections. To characterize the response, we measured the average pairwise correlation of the spiking activities (binning window of 20 ms) within each MSN group (*W*_*out*_) and between the two MSN groups (*B*_*out*_). Thus, correlation transfer denotes the mapping from effective input correlations ($W_{\text{in}},B_{\text{in}}*W_{\text{in}}$) to output correlations ($W_{out},B_{out}$) (see Input model).

**Fig 3:**
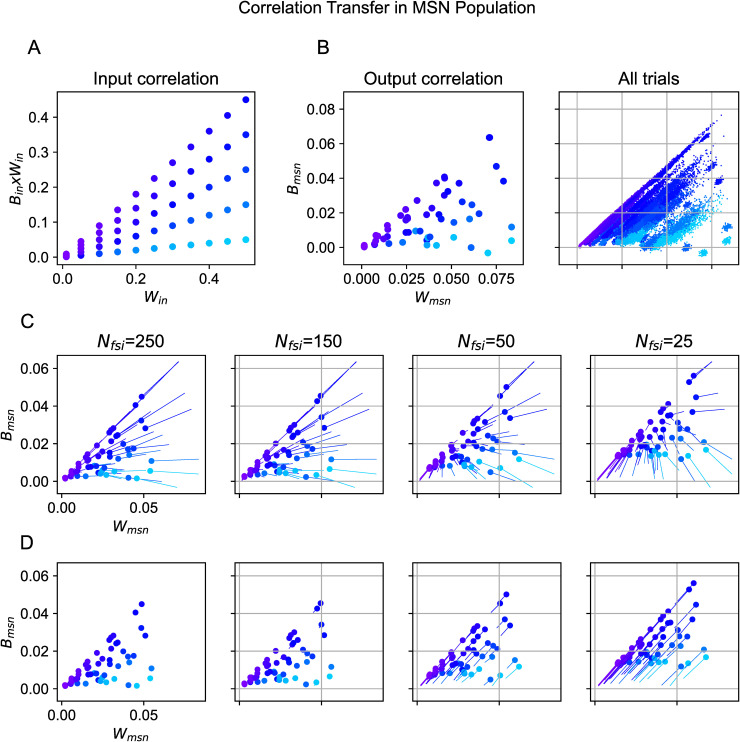
Modulation of correlation by sharing of FSIs in the striatum during evoked states. **(A)** Effective correlation settings for the cortical inputs. **(B)** Output statistics of MSNs for *N*_fsi_ = 0. Left: output correlation (averaged across 100 trials for each input setting) of MSNs’ spiking activity. The x-axis denotes mean pairwise correlation within the MSN group (*W*_msn_) while the y-axis denotes mean pairwise correlation between two MSN groups (*B*_msn_). The color corresponds to **A**. Right: all trials of output correlations. **(C)** Each subplot compares the correlation of MSN activities between the control case (no FSI) and the studied case (a certain number of FSIs or degree of FFI sharing). Each line corresponds to a particular input correlation (color code same as in panel **A**). The starting and ending points of the lines denote the trial-averaged output correlation for *N*_fsi_ = 0 and *N*_fsi_ > 0, respectively. From left to right, the value decreased from *N*_fsi_ = 250 to *N*_fsi_ = 25 (or the sharing of FFI increased). **(D)** Same as in panel **C**, but in this case the starting point of each line is the output correlation measured for *N*_fsi_ = 250.

Because we were interested in isolating the role of FSIs in modulating the correlation, we compared the output correlation under different sharing levels while keeping the input fixed. To this end, we first generated cortical inputs with a wide range of sharing levels (see Input model, Fig 3A). Given noisy backgrounds across trials, the output correlation fluctuated even in the baseline case without any FSI (Fig 3B Right). Therefore, we used the trial-averaged values for the transferred output correlations (Fig 3B Left).

In Fig 3C, we rendered the modulation of correlation in the presence of FSIs compared to the baseline case when there were no FSIs. When FSI inputs were weakly shared (*N*_fsi_ = 250), feedforward inhibition decorrelated the MSN activity (compared to the baseline case of no FSIs) for all input configurations (Fig 3C Left). However, when FSIs were highly shared (*N*_fsi_ = 25) as in the striatum, MSN synchrony could be both increased or decreased (compared to the baseline case of *N*_fsi_ = 0) depending on the inputs (Fig 3C Right). The effect of sharing of FSIs became more apparent when we compared the MSNs correlation for *N*_fsi_ = 250 with *N*_fsi_ = 25 (Fig 3D). For all input settings, MSNs were more correlated when there were fewer FSIs.

The increase in output correlations because of shared FSIs was not trivial, as the output firing rates had minimal differences comparing the two cases *N*_fsi_ = 25 and *N*_fsi_ = 250. Furthermore, there was no relationship between rate modulation and correlation modulation (see S3 Fig). In the simulations above, we assumed that all neurons of a given type had the same parameters and that synaptic weights were drawn from a lognormal distribution. Therefore, we can argue that the correlating effect of FSI inhibition arose from the FFI sharing and would only manifest for highly shared projections, which in turn creates a bidirectional modulation of correlation in the striatum.

To test whether the results are affected by neuron heterogeneity, next, we introduced heterogeneity among neurons (see Neuron model). Neuronal heterogeneity reduced the correlation by a small amount without changing the qualitative results S4 Fig. Still, *N*_fsi_ = 25 induced more correlation than *N*_fsi_ = 250 (S5B Fig Right). With neuron heterogeneity, FFI sharing also affected MSN firing rate, but there was no clear relationship with the input correlations (S5A Fig Right). Curiously, the change in MSN firing rate due to FFI sharing was correlated with the change in output correlations among MSNs (S5D Fig). This rate-correlation effect is consistent with the idea that firing rates increase correlation transfer [13].

### Effect of FFI sharing on downstream GPe activity

Next, to test whether and how the increase in across-trial variability and correlation may affect the downstream neurons in the globus pallidus (GP), we simulated a single neuron. This neuron was innervated by excitatory background noise such that it spiked at a rate comparable to GPe neurons (i.e., 40 Hz [20]) and was inhibited by the projections from MSNs (see Downstream effect). As before, we took the response of GPe for *N*_fsi_ = 250 as our baseline and systematically reduced the number of FSIs while keeping other parameters unchanged.

**Fig 4:**
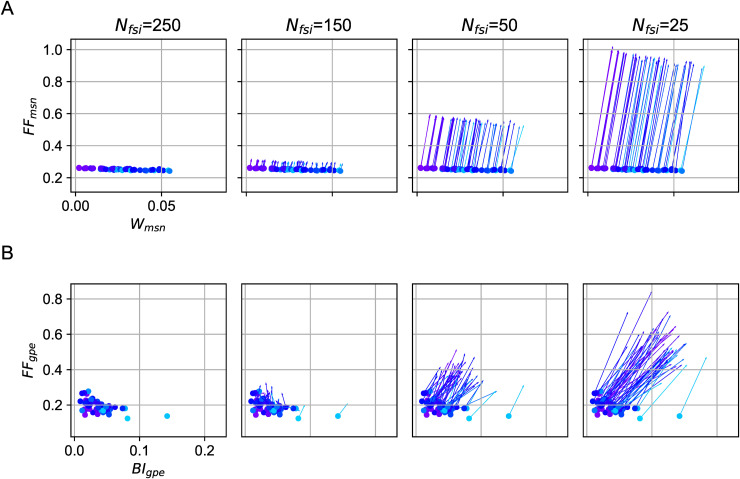
Downstream effect of FFI sharing on a GPe neuron. **(A)** We describe the state of MSN population activity as average pairwise correlation (x-axis) and across-trial variability (y-axis). Each line starts at a point indicating average pairwise correlation and across-trial variability for $25\leq N_{\text{fsi}}<250$ and ends at a point indicating average pairwise correlation and across-trial variability for *N*_fsi_ = 250. A filled circle marks the ending of a line. Each line corresponds to a specific input correlation (see Fig 3A). The correlation level and across-trial variability of population firing increase with stronger sharing of feedforward inhibition (*N*_fsi_ changing from 250 to 25). **(B)** The bursting index (x-axis) and across-trial variability (y-axis) of a GPe neuron in response to MSNs’ activity during evoked states. Each line starts at a point indicating average bursting index correlation and across-trial variability for $25\leq N_{\text{fsi}}<250$ and ends at a point (marked with a filled circle) indicating average bursting index and across-trial variability for *N*_fsi_ = 250.

With *N*_fsi_ = 25, MSN activities were more correlated within trials, and more variable across trials than with *N*_fsi_ = 250 (Fig 4A Right). The statistics of MSN activity were reflected in the GPe firing rate distribution across trials and burstiness within trials, respectively (Fig 4B Right; see S6 Fig and Downstream effect for detailed definitions). Both across-trial variability and burstiness increased as we decreased the number of FSIs (Fig 4B, left to right). However, there was no significant relationship between the rate modulation and either the across-trial variability or the burstiness of spiking activities (see S7 Fig). These results suggest that the effect of FFI sharing in the striatum has a meaningful impact on the firing of GPe, especially on bursting, which has clear functional correlates [21].

These results remained unchanged when we considered a model where both neuron parameters and synaptic weights were chosen from a probability distribution (see S1 Fig). As we saw above, in the presence of neuron heterogeneities, FFI sharing affected MSN firing rates and correlations S5 Fig. The rate effect was also transferred to the GPe neuron in the form of a correlation between FSI-induced rate difference and bursting index of the GPe neuron S8D Fig.

## Discussion

The striatum is a network in which a minimal number (only about 1%) of FSIs provide feedforward inhibition from the cortex to MSNs (95%). Axonal morphology of FSIs allows them to target a larger number of MSNs. Thus, unlike in the neocortex, feedforward inhibition in the striatum is highly shared among MSNs. It is already known that such sharing of FSIs can induce synchrony in the striatum, which can be countered by the recurrent inhibition of MSNs [11,22]. We used a computational model to reveal two more consequences of such connectivity of FSIs. We show that shared FSIs (1) generate high across-trial variability at the population level and (2) provide a bidirectional modulation of correlation during evoked activity.

The results arise from the sharing of inputs and Dale’s law, i.e., all FSIs have an inhibitory effect on the MSNs and do not depend on the actual operating point of the network. To understand this, consider two scenarios: when MSNs receive high or low sharing of FSI projections (Fig 1C). Because each MSN receives the same number of inputs in the two scenarios, the activity of individual MSNs is similar in the two scenarios. However, the correlation between MSN activities depends on the level of FFI sharing. As long as all FSIs hyperpolarize the MSNs (i.e., FSIs follow Dale’s Law), synaptic strengths and neuron excitabilities can only alter the degree of correlation but not the sign of correlation. Therefore, we argue that neuron and synapse properties can only change our results quantitatively, not qualitatively. We have confirmed it in simulations where both neuron and synapse properties were drawn from a distribution (S2 and S4 Figs). In short, when FSIs are shared, the subthreshold membrane potential of MSNs becomes correlated, leading to high variance at the population level and high across-trial variability.

As said, our model predicts that high sharing of FSI inputs should induce high across-trial variability and within-trial correlation of MSN outputs. However, there are only a few reports on task-related increases in synchrony among MSNs [23]. There are several possibilities for how, despite shared FSI inputs, MSNs can maintain low synchrony. First, recurrent inhibition among MSNs can weaken the synchrony [11]. However, this effect is likely small given the weak recurrent inhibition among MSNs [9]. There is another potential mechanism that can desynchronize MSNs. Depending on the reversal potential, the GABAergic input may also be excitatory [24]. Therefore, a strong decorrelation may occur if different MSNs show a wide range of mean membrane potential such that FSIs’ inputs appear excitatory on some MSNs. Lastly, neuromodulators can also weaken the activities of some FSIs, reducing the effective sharing of FFI [25].

Here, we have ignored any connectivity between FSIs. Experimental data suggest that FSIs may be recurrently connected via chemical or gap junctions. While gap junctions tend to synchronize FSIs [26,27], inhibitory chemical synapses could potentially desynchronize the FSI activity. Thus, the presence of both chemical and electrical synapses may change the effective correlation of FFI inputs [28]. Our results show that FSIs themselves increase the correlation of MSN activities even if they themselves are uncorrelated. If any other process, such as gap junctions, increases the correlations among FSIs, then FSIs would induce stronger dynamical effects.

### Functional consequence

**Fig 5:**
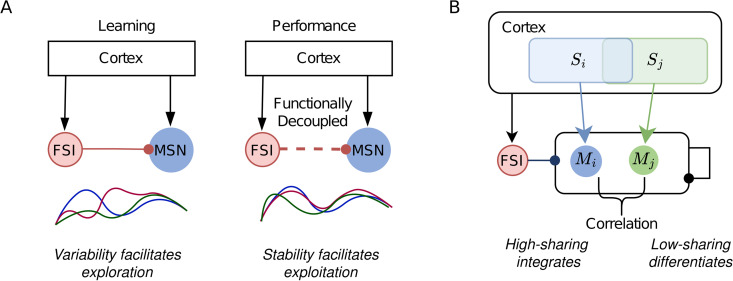
Possible functional consequences of FSI modulations. **(A)** During the learning phase, the high-sharing of FSI induces variable population responses across trials, which facilitates exploration behavior; during the performance phase, FSIs are functionally decoupled, leading to stable responses across trials, which facilitates exploitation behavior. **(B)** Different groups of MSNs receive cortical inputs from distinct but overlapped sources. In contrast to MSNs, FSIs receive inputs from both sources. Whether the signals get integrated (high correlation between the activities of two MSN groups) or differentiated (low correlation) is modulated by the sharing level of FSI projections to MSNs.

Across-trial neural variability is a fundamental characteristic of healthy brain function, where abnormal variability can disrupt cognitive processes [29]. We propose that FSIs may play a regulatory role in learning processes by modulating across-trial variability, as illustrated in Fig 5A. During learning phases, elevated variability mediated by FSIs could facilitate exploratory behavior by generating diverse neural activities. However, during task performance, FSIs may become functionally decoupled from MSNs to minimize variability and stabilize learned behaviors. This hypothesis aligns with experimental findings showing that optogenetic silencing of FSIs impairs acquisition of striatum-dependent egocentric learning while leaving performance of established motor behaviors intact [15]. The proposed dynamic engagement of FSIs is further supported by evidence that these interneurons do not uniformly suppress MSN activity [30], suggesting the existence of mechanisms for functional disconnection selectively. Such flexible modulation of FSI-MSN interactions could enable the striatum to alternate between exploratory and stable operational modes as behavioral demands require.

The bidirectional modulation of correlation provides a potential mechanism for regulating signal integration and discrimination where neuron density and properties show spatial gradients (Fig 5B). For highly shared inputs (large *B*_*ij*_), MSN groups become decorrelated due to recurrent inhibition; for weakly shared inputs (small *B*_*ij*_), MSN groups become correlated due to shared inhibition from FSIs. The threshold of *B*_*ij*_ below which FSIs correlate the activity can be dynamically adjusted by changing the degree of FFI sharing or, more easily, by modulating the strength of FSI projections, offering a possible mechanism for how different cortical sources get correlated/decorrelated at the striatum. While direct experimental evidence for FSI-mediated multi-modal integration and discrimination remains limited, the anatomical observation supports this possibility. FSIs receive convergent inputs from broad cortical regions spanning multiple sensory and motor areas [31], positioning them ideally to facilitate cross-modality integration. This widespread connectivity pattern suggests FSIs may serve as a neural substrate for sensorimotor coordination, potentially enabling the striatum to combine information from distinct cortical streams through precisely controlled inhibitory synchronization. As reported in [32,33], the latency of feedforward inhibition to feedforward excitation depends on the stimulus condition. The heterosynaptic delays of all connections (see Connectivity model) in our simulations suggest that the correlating effect could be robust to temporal delays between cortical sources as well as between FFE and FFI.

Finally, FSIs themselves are not uniformly distributed in the striatum, with a ventromedial to dorsolateral gradient [34,35] in the dorsal striatum and with varied abundance in the caudate nucleus and putamen [36]. Therefore, the functional consequences of FSI could be specific to the subregion. The distinct density of FSIs would lead to different levels of across-trial variability and correlations as shown in Fig 2 and 3. Since the striatum is known to topographically receive and reflect cortical activities [14,37], it would be intriguing to investigate whether the distribution of FSI is aligned with the modality of cortical inputs.

### Experimental falsification

We predict that shared FSI inputs can increase across-trial variability and modulate correlation transfer. However, these predictions cannot be falsified by simply silencing the FSIs – ablation of FSIs will remove the feedforward inhibition and increase the MSN firing rate [15]. On the other hand, artificially increasing the firing rate of FSIs will decrease the MSN firing rate, even though without altering FFI sharing. To check the predictions, the connectivity structure needs to be changed. For instance, there is an increase in both the correlation between MSNs and the sharing of FSI projections to MSNs [22,38] in Parkinson’s disease, which is consistent with our model; however, how the across-trial variability changes remains to be experimentally checked.

Another way to falsify our predictions is by patching two MSNs. According to our results, FSIs will give rise to subthreshold synchrony in their membrane potential. If we alter the mean membrane potentials of the pair of neurons such that FSI inputs could evoke depolarization in one and hyperpolarization in another. The subthreshold synchrony between the two neurons should decrease. A caveat of this manipulation is that it may also alter the sign of MSN inputs. However, MSN inputs are mostly on dendrites, and FSI inputs are on soma; therefore, changing somatic membrane potential may not affect the sign of MSN inputs in the dendrites. To our knowledge, simultaneous dual patching has not been performed in the striatum of awake animals, though it has already been done in anesthetized animals [39].

### Functional role of small neural populations

In many regions of the brain, there are neuron types that comprise only a small fraction of the whole population, e.g., different types of interneurons in the striatum and several types of neurons in the hypothalamus [40]. It raises questions about how a neuron type affects the network activity given a certain connection probability and relative size (Fig 1B). “Rare” neuron types can affect the activity and function of a network in two ways: (1) by connecting with a specific pattern, and (2) by connecting with all neurons within the axonal reach. In the latter case, regardless of whether they form excitatory or inhibitory synapses, our work suggests that such rare types of neurons should affect the network dynamics in terms of across-trial variability and pairwise correlation.

## Methods

### Neuron model

We implemented all neurons using conductance-based leaky integrate-and-fire models [41], with distinct parameter sets for medium spiny neurons (MSNs), fast-spiking interneurons (FSIs), and the Globus Pallidus externa (GPe) neuron as specified in Table 1. The subthreshold membrane potential dynamics of each MSN followed the differential equation:


$$\begin{array}{cc} {\text{C}}_{m}\frac{dV_{\text{msn}}}{dt} & =-{\text{g}}_{L}(V_{\text{msn}}-{\text{E}}_{L})\\ & -g_{e}^{\text{bkg}}(t)(V_{\text{msn}}-{\text{E}}_{e})-g_{e}^{\text{ctx}}(t)(V_{\text{msn}}-{\text{E}}_{e})\\ & -g_{i}^{\text{msn}}(t)(V_{\text{msn}}-{\text{E}}_{i})-g_{i}^{\text{fsi}}(t)(V_{\text{msn}}-{\text{E}}_{i}) \end{array}$$ (1)

where *V*_msn_ represents the membrane potential, Cm the membrane capacitance, gL the leak conductance, and EL the resting potential. The model incorporated four types of synaptic inputs (see Fig 1A), each modeled as conductance-based synaptic events: (1) $g_{e}^{\text{bkg}}(t)$ for background excitation, (2) $g_{e}^{\text{ctx}}(t)$ for cortical excitation, (3) $g_{i}^{\text{msn}}(t)$ for recurrent inhibition from other MSNs, and (4) $g_{i}^{\text{fsi}}(t)$ for feedforward inhibition from FSIs. When a neuron from the presynaptic source spikes, it sends the signal to the postsynaptic neuron as one of the four types. The dynamic of synaptic conductance is given in Eq. 2. When the membrane potential reached the threshold V_th_, the neuron fired a spike and reset to V_reset_, with a subsequent refractory period *t*_r_. FSIs followed similar dynamics but lacked inhibitory inputs, reflecting their position as the primary inhibitory source in the network. GPe only receives background and MSN innervations.

**Table 1:** Neuron parameters.

	C_m_ (pF)	*g*_L_ (nS)	E_L_ (mV)	V_th_ (mV)	V_reset_ (mV)	*t*_r_ (ms)	*E*_e_ (mV)	*E*_i_ (mV)	$\tau_{\text{e}}$ (ms)	$\tau_{\text{i}}$ (ms)
**MSN**	80	10	-80	-45	-70	2	0	-85	0.2	15
**FSI**	70	5	-70	-40	-60	2	0	-85	0.2	15
**GPe**	70	2.5	-70	-45	-60	2	0	-85	0.2	15

We used different neuronal parameters in accordance with the typical firing rates of MSNs, FSIs, and GPe neurons. Given the parameters in Table 1, MSNs have a rheobase of current injection at around 320 pA, FSIs around 140 pA, and GPe around 60 pA (see S1A Fig).

All results shown in the main text are for homogeneous neurons. In S2, S4, S5 and S8 Figs, we show the results for heterogeneous neurons. In those simulations, we introduced heterogeneous neuronal properties such that ${\text{C}}_{m},g_{\text{L}},{\text{V}}_{\mathrm{th}},t_{\text{r}}$ were all sampled independently with a normal distribution of corresponding mean in Table 1, and variance equal to 1/30 of the mean. In turn, almost all values lie within a relative range of ±10% ($3\sigma =\frac{3}{30}$). The F-I curves of heterogeneous neuronal properties are shown in S1A Fig.

### Synapse model

The neurons were connected using conductance-based synapses. Each incoming spike resulted in a conductance transient *g*_syn_(*t*) following an alpha function with a time constant $\tau_{\text{syn}}$. The equation described the synaptic dynamics:


$$g_{\text{syn}}(t)=\sum_{i}\overset{―}{g_{\text{syn}}}\frac{t-t_{i}}{\tau_{\text{syn}}}exp(-\frac{t-t_{i}}{\tau_{\text{syn}}})H(t-t_{i})$$ (2)

where *t*_*i*_ is the arrival time of *i*^th^ spike and *H* is the Heaviside step function. The four types of synaptic inputs to the MSN in Eq. 1 all follow this synaptic dynamic.

The time constants for excitatory and inhibitory synapses, $\tau_{\text{e}}$ and $\tau_{\text{i}}$, are given in Table 1. Since FSI inhibition is dominated by GAB_AA_ receptors [10] with a time constant in the scale of 10 ms, we used 15 ms for $\tau_{\text{i}}$.

The peak conductance for each synaptic type is given by $\overset{―}{g_{\text{syn}}}$. All synaptic strengths (peak conductances) were independently sampled from a lognormal distribution of σ=0.5 with corresponding averages listed in Table 2. Distributions for inhibitory synapses are shown in S1B Fig. The synaptic delays denote the transmission time of a spike from a presynaptic neuron to a postsynaptic neuron. They were chosen to be relatively fast (1 ms) for excitatory synapses and slow (2 ms) for inhibitory synapses. All synaptic delays were uniformly sampled from ±1 ms of the corresponding values in Table 2.

**Table 2:** Network connectivity parameters.

		Cond.	(nS)			Delay	(ms)	
Tar\Src	Bkg.	Ctx.	FSI	MSN	Bkg.	Ctx.	FSI	MSN
FSI	1.0	0.5(0.25)	–	–	1	1	–	–
MSN	2.0	4.8	0.5	0.03	1	1	2	2

The heterogeneous synaptic weights and delays were used in all simulations. The time constant of inhibitory synapses was sampled from a normal distribution with mean $\tau_{\text{i}}=$ 15 ms and variance of value 0.5 for the heterogeneous neuron case.

### Connectivity model

The network architecture for all simulations of striatal microcircuits maintained the basic structure shown in Fig 1A, comprising 2,500 medium spiny neurons (MSNs) with a fixed indegree of 250 lateral inhibitions (autapse allowed). For all connections, multapses are permitted. To investigate how feedforward inhibition (FFI) sharing affects network dynamics, we systematically changed the number of fast-spiking interneurons (FSIs) while maintaining a constant number of FSI inputs ($g_{\mathrm{in}}^{\text{FSI}}=15$) to each MSN. This experimental design created a range of FFI sharing conditions by changing the density (size) of the FSI population ($\frac{g_{\mathrm{in}}^{\text{FSI}}}{N_{\text{FSI}}}$). We systematically changed it from highly shared (60% when only 25 FSIs provided inputs) to minimally shared (6% with 250 FSIs), with a complete absence of FSIs serving as the control condition.

The synaptic parameters governing connection strengths ($\overset{―}{g_{\text{syn}}}$) and transmission delays are listed in Table 2, with several biologically motivated constraints incorporated into the network architecture. Experimental observation showed that the somatic effect of FSI inhibition is much stronger than the recurrent inhibition between MSNs [10], so we adopted a strong FSI-MSN connection and a weak MSN-MSN connection. There was no feedback projection from MSNs to FSIs [42]. The absence of FSI-FSI connections represents a conservative approach, as such connections primarily serve to synchronize interneuron activities [26,27]. The increased synchrony would increase the effective sharing of FFI, which is already captured by the FFI sharing parameter in our model. We reduced the synaptic strength of cortical projections to FSIs by 50% (to 0.25) to account for convergent inputs from both cortical sources.

### Spontaneous activity

To simulate ongoing background activity in the network, we implemented independent Poisson-type excitatory inputs to each neuron. MSNs received baseline inputs at 5.95 kHz (reduced to 3.2 kHz in simulations without FSIs to maintain consistent firing rates), while fast-spiking interneurons (FSIs) received inputs at 5.75 kHz. These background inputs served to maintain physiologically realistic spontaneous firing rates of approximately 1 Hz for MSNs and 7 Hz for FSIs. The synaptic weights for these background projections were tuned (as specified in Table 2) to achieve these target firing rates.

Given that MSN and FSI activities are largely independent in the ongoing state [17], it is reasonable to assume independent background input to the MSNs and FSIs. In addition, there is no strong evidence as to whether MSNs are correlated or not when receiving background drive from the cortex. Therefore, we did not add any correlation in the baseline inputs.

### Input model

To mimic the corticostriatal projections, we used a connection setting as illustrated in Fig 1C Right. Each cortical neuron generated independent Poisson spike trains that propagated to downstream striatal targets. Within each MSN group (represented by blue and green populations), divergent cortical connections created shared inputs of spike trains that would induce pairwise correlations. We quantified this input sharing within each MSN group as $W_{\text{in}}=g_{\mathrm{in}}^{\text{ctx}}/N_{\text{ctx}}$, where $g_{\mathrm{in}}^{\text{ctx}}=100$ denotes the fixed indegree of cortical inputs and *N*_ctx_ denotes the population size of cortical sources. Changing the population size of cortical sources changes the level of input sharing without varying the total cortical inputs. On top of it, we could differentiate the MSNs into two groups. The two MSN groups received cortical inputs from overlapping sources. As illustrated, the input sharing between two groups was quantified as $B_{\text{in}}=N_{\text{ol}}/N_{\text{ctx}}$, where *N*_ol_ is the number of overlapping cortical neurons and *N*_ctx_ is the population size of each cortical source. Similarly, changing the population size and overlapping size changes the level of input sharing between the two MSN groups; hence, the effective between-group correlation is $B_{\text{in}}*W_{\text{in}}$. In short, the parameters *W*_in_ and *B*_in_ represent the sharing of feedforward excitation (FFE).

Note that, when *W*_in_ is small, feedforward excitations are sampled from a large cortical population; there are very few multapses. When *W*_in_ increases, the number of multapses increases, effectively inducing correlated inputs to individual MSNs. As shown in Fig 2C, the firing rate of MSNs increased with *W*_in_ due to more multapses. When multapses are forbidden, the output rate would remain almost the same across all input sharing settings (as a minor point, not included in the paper).

Similarly, FSIs received cortical projections from both cortical sources with a fixed indegree of $g_{\mathrm{in}}^{\text{ctx}}=100$. In turn, the neurons in the overlapped part of cortical sources have a double chance of connections with each FSI. Hence, the synaptic strength is half compared to the cortical projections to MSNs (see Table 2).

### Stimulus-evoked activities

The strengths of cortical inputs were set to achieve a steady-state firing rate of approximately 5 Hz for MSNs and 17 Hz for FSIs during evoked states. We kept the rate of cortical spike trains fixed at 10 Hz, and systematically changed the input sharing within-group *W*_in_ ([0.01 0.05 0.1 0.15 0.2 0.25 0.3 0.35 0.4 0.45 0.5]) and between-group *B*_in_ ([0.1 0.3 0.5 0.7 0.9]). The two MSN groups received cortical inputs from two partially overlapped sources, while the FSI population received inputs from both cortical sources.

For each combination of FFE and FFI sharing parameters, we conducted 100 independent simulation trials lasting 2.5 seconds. The initial 500 ms of each trial allowed network activity to stabilize, with the subsequent 2-second period used for statistical analysis. Throughout these repeated trials, the cortical inputs remained identical, while the background noise was independently generated.

## Statistical analysis

### Across-trial variability

Across-trial variability reflects the consistency of population activity in response to the same stimulus, given a noisy background in different trials. We quantified it with the Fano factor of the population rate across trials. For each time bin of 2 ms:


$$F_{i}=\frac{\sigma^{2}(r_{i}{)}_{\text{trial}}}{\mu (r_{i}{)}_{\text{trial}}}$$

where *r*_*i*_ is the population-averaged firing rate of MSNs within the corresponding time bin. The calculation was performed for the last 2 s of the simulation and averaged across time bins as the variability index *FF*_MSN_. The variability index reflects the temporal variability of the population rate across trials. It differs from the synchrony index defined as the Fano factor of population rate within a trial [11]. Both the within-trial synchrony and the across-trial variability originate from the sharing of FSI inhibition. The shared FSI inputs induce high covariance between neurons for synchrony and amplify the effect of shared noise for across-trial variability.

### Correlation transfer

The correlation transfer denotes the transformation from the effective input correlations $\left(W_{\text{in}},B_{\text{in}}*W_{\text{in}}\right)$ to the output correlations $\left(W_{\text{out}},B_{\text{out}}\right)$, as shown in Fig 1C. The output correlations were measured as the average pairwise correlation coefficient of the spiking activities within each group *W*_out_ and between the two groups *B*_out_. The correlation coefficient is calculated with a 20 ms binning of the spike train between any pair of MSNs:


$$R_{ij}=\frac{C_{ij}}{\sqrt{C_{ii}C_{jj}}}$$

where *C*_*ij*_ is the covariance between the pair of spike trains. Therefore, the output correlation is defined as the averages across pairs:


$$\begin{array}{cc} W_{\text{out}} & =\frac{1}{N_{\text{MSN}}^{a}(N_{\text{MSN}}^{a}-1)}\sum_{\begin{array}{c} i<j\\ i,j\in M^{a} \end{array}}R_{ij}\;+\;\frac{1}{N_{\text{MSN}}^{b}(N_{\text{MSN}}^{b}-1)}\sum_{\begin{array}{c} i<j\\ i,j\in M^{b} \end{array}}R_{ij}\\ B_{\text{out}} & =\frac{1}{N_{\text{MSN}}^{a}\;N_{\text{MSN}}^{b}}\sum_{\begin{array}{c} i\in M^{a}\\ j\in M^{b} \end{array}}R_{ij} \end{array}$$

### Downstream effect

We simulated one GPe neuron that received excitatory background noise (a Poisson spike generator of 7k Hz) and feedforward inhibition from one MSN population (1250 MSNs). The MSN projections follow a lognormal distribution of strength (mean 0.02 nS, variance 0.5, see S1B Fig), and a uniform distribution of delays (1–3 ms). In turn, the GPe neuron fired in the range of 20–50 Hz, consistent with the extreme firing level of GPe neurons *in vivo* [20], under different sharing settings of cortical projections to the striatum.

Across trials, we measured the variability of GPe firing as the Fano factor of spike count across trials:


$$FF_{\text{GPe}}=\frac{\sigma^{2}(N_{\text{spike}}{)}_{\text{trial}}}{\mu (N_{\text{spike}}{)}_{\text{trial}}}$$

Within trials, we measured the bursting index of GPe firing as the proportion of bursting spikes averaged across trials, where a burst is a group of consecutive spikes with size larger than three and inter-spike interval smaller than 10 ms.


$$BI_{\text{GPe}}=\mu (\frac{N_{\text{spike}}^{\mathrm{burst}}}{N_{\text{spike}}^{\mathrm{total}}}{)}_{\text{trial}}$$

### Simulation and data analysis tools

Simulations were performed using the NEST simulator [43]. The differential equations were integrated using a fixed time step of 0.1 ms. The analysis of simulated network activity was done using customized code written in Python. The results were visualized using Matplotlib.

### Code availability

The code for simulation and plotting is shared on GitHub https://github.com/weirdoglh/FSInet.

## Supporting information

S1 Fig Heterogeneous F-I curves and inhibitory synaptic strengths. (TIFF)

S2 Fig Across-trial variability of MSN activities with heterogeneous neurons. (TIFF)

S3 Fig Relationship between output rate and output correlation of MSNs with homogeneous neurons. (TIFF)

S4 Fig Modulation of correlation by sharing of FSIs in the striatum with heterogeneous neurons. (TIFF)

S5 Fig Relationship between output rate and output correlation of MSNs with heterogeneous neurons. (TIFF)

S6 Fig Spiking statistics of the GPe neuron during evoked states. (TIFF)

S7 Fig Relationship between output rate and spiking statistics of GPe with homogeneous neurons. (TIFF)

S8 Fig Relationship between output rate and spiking statistics of GPe with heterogeneous neurons. (TIFF)
