# Supplementary figures and images for: Role of fast-spiking interneurons in modulating across-trial variability and within-trial correlations in the striatum
Source: PLoS Comput Biol. 2026 Mar 27;22(3):e1014099. doi: 10.1371/journal.pcbi.1014099 (PMC13095104; doi:10.1371/journal.pcbi.1014099)

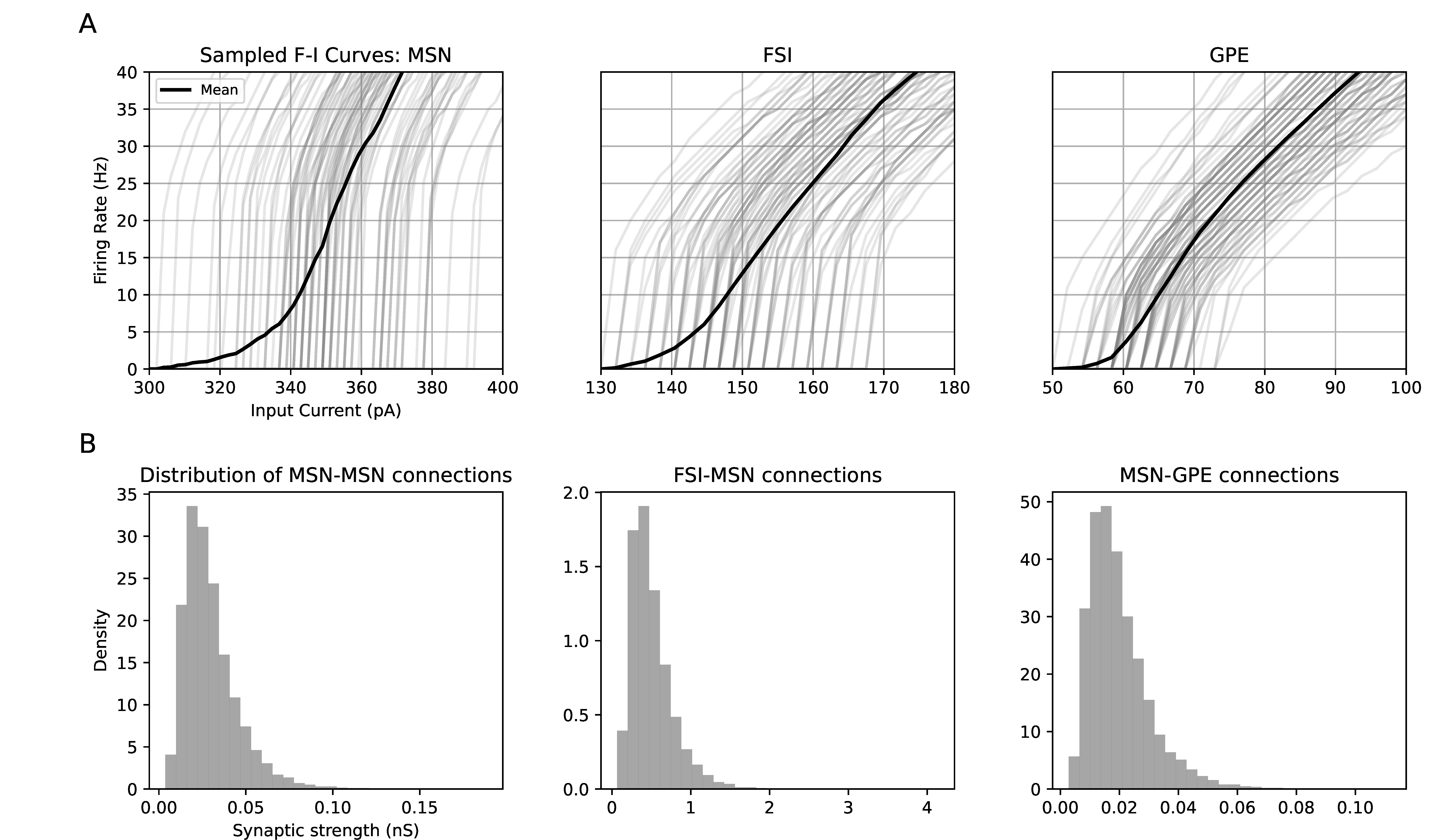

Supplement: S1 Fig — (TIFF) [file pcbi.1014099.s001.tiff]

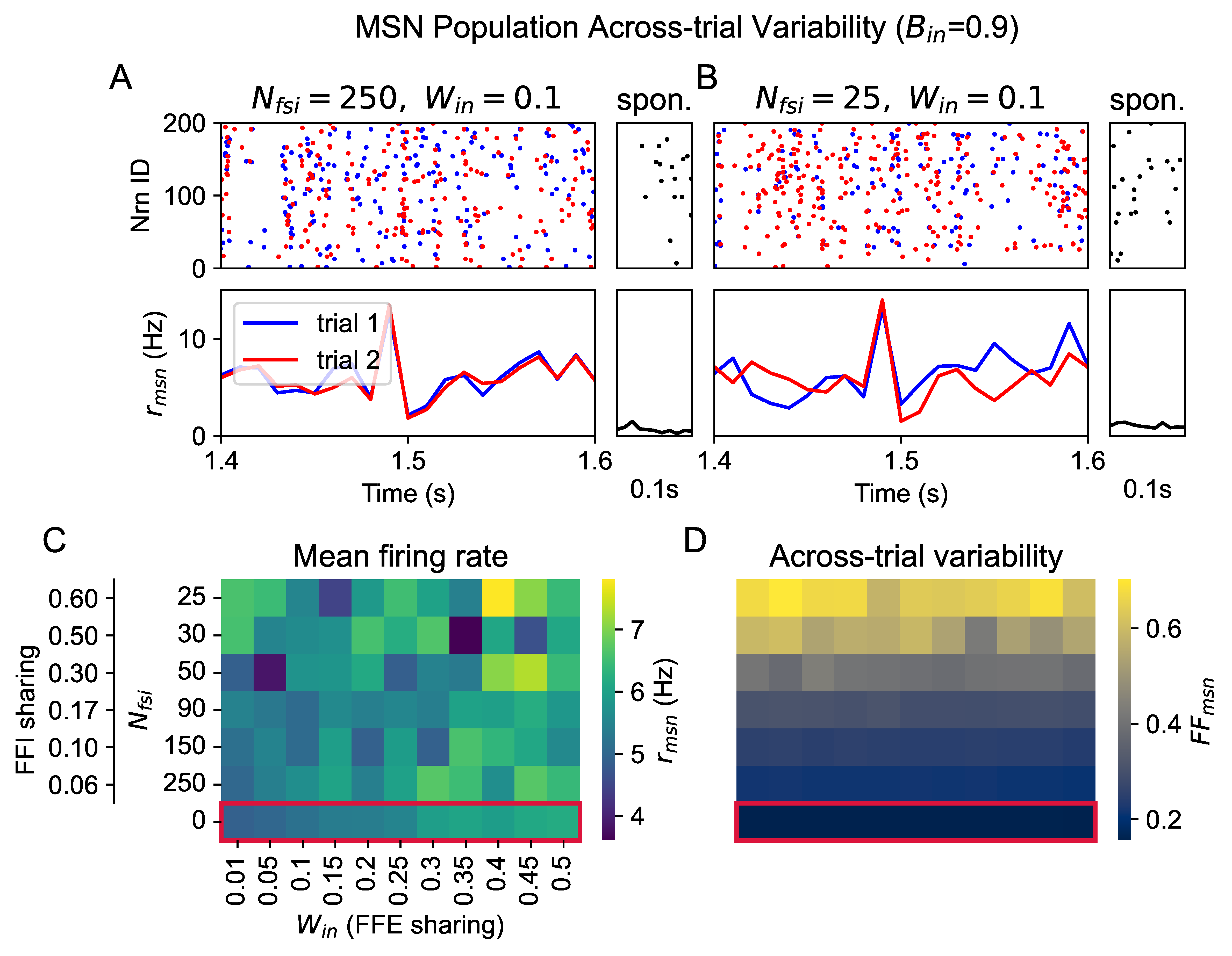

Supplement: S2 Fig — (TIFF) [file pcbi.1014099.s002.tiff]

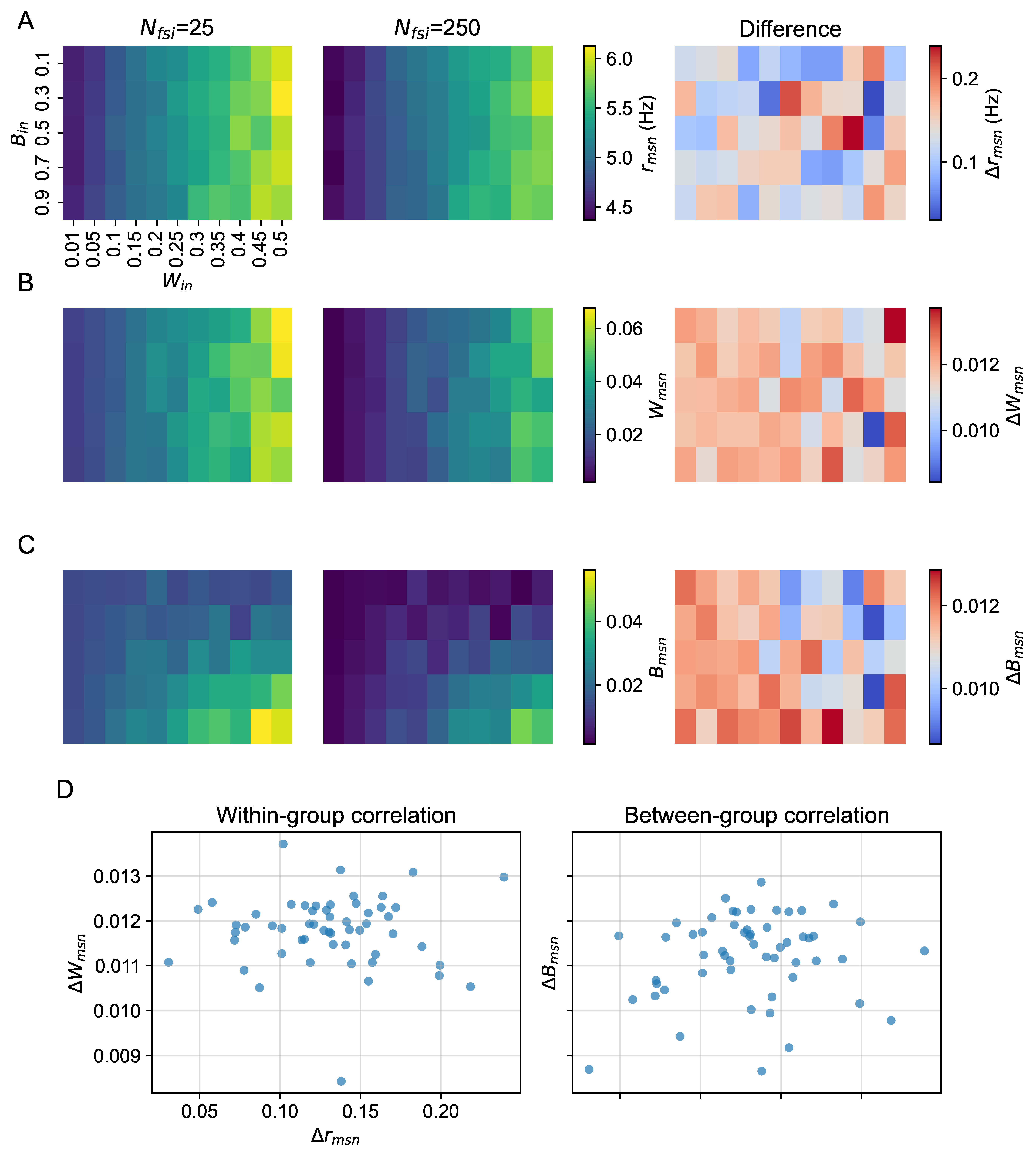

Supplement: S3 Fig — (TIFF) [file pcbi.1014099.s003.tiff]

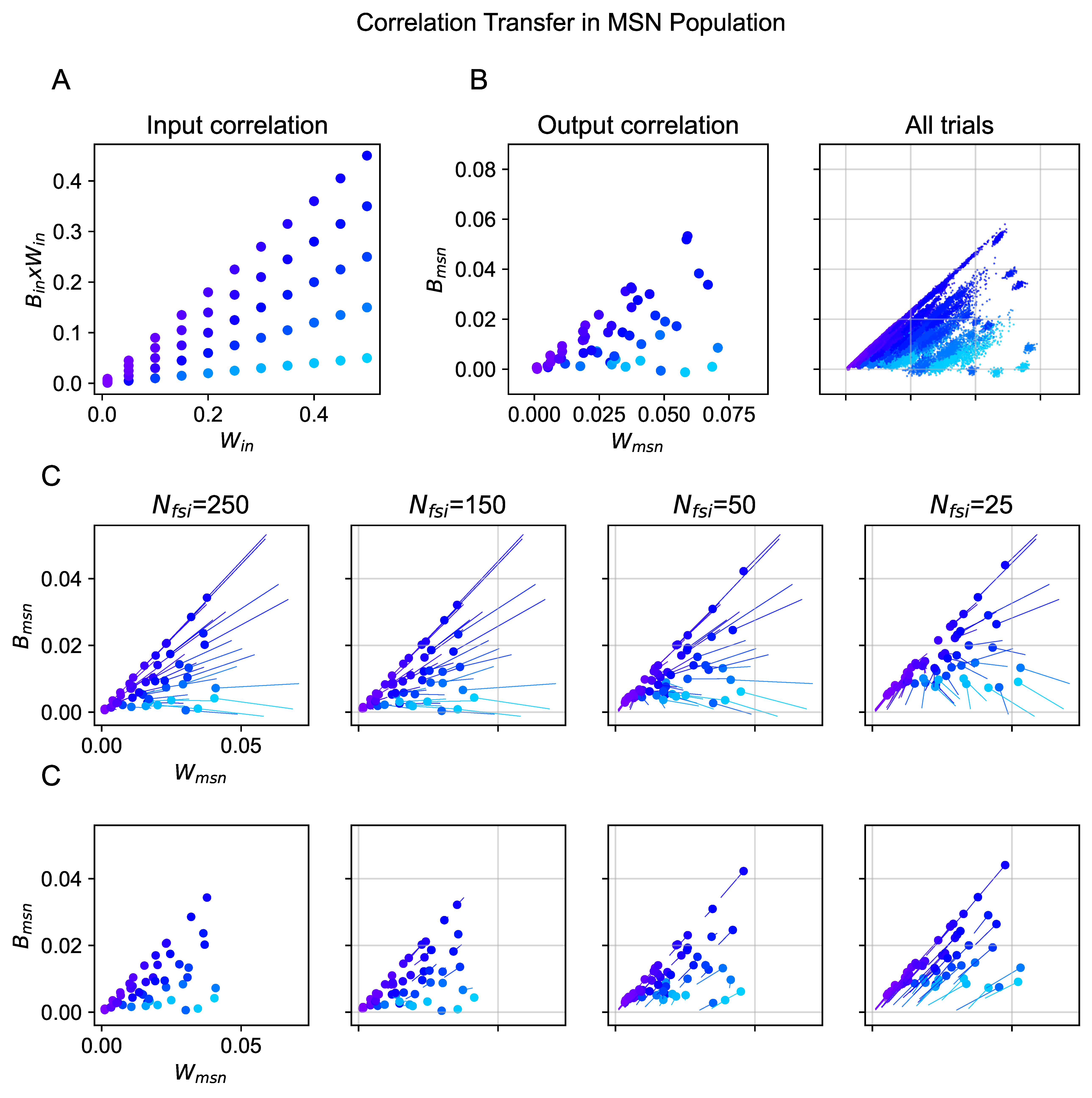

Supplement: S4 Fig — (TIFF) [file pcbi.1014099.s004.tiff]

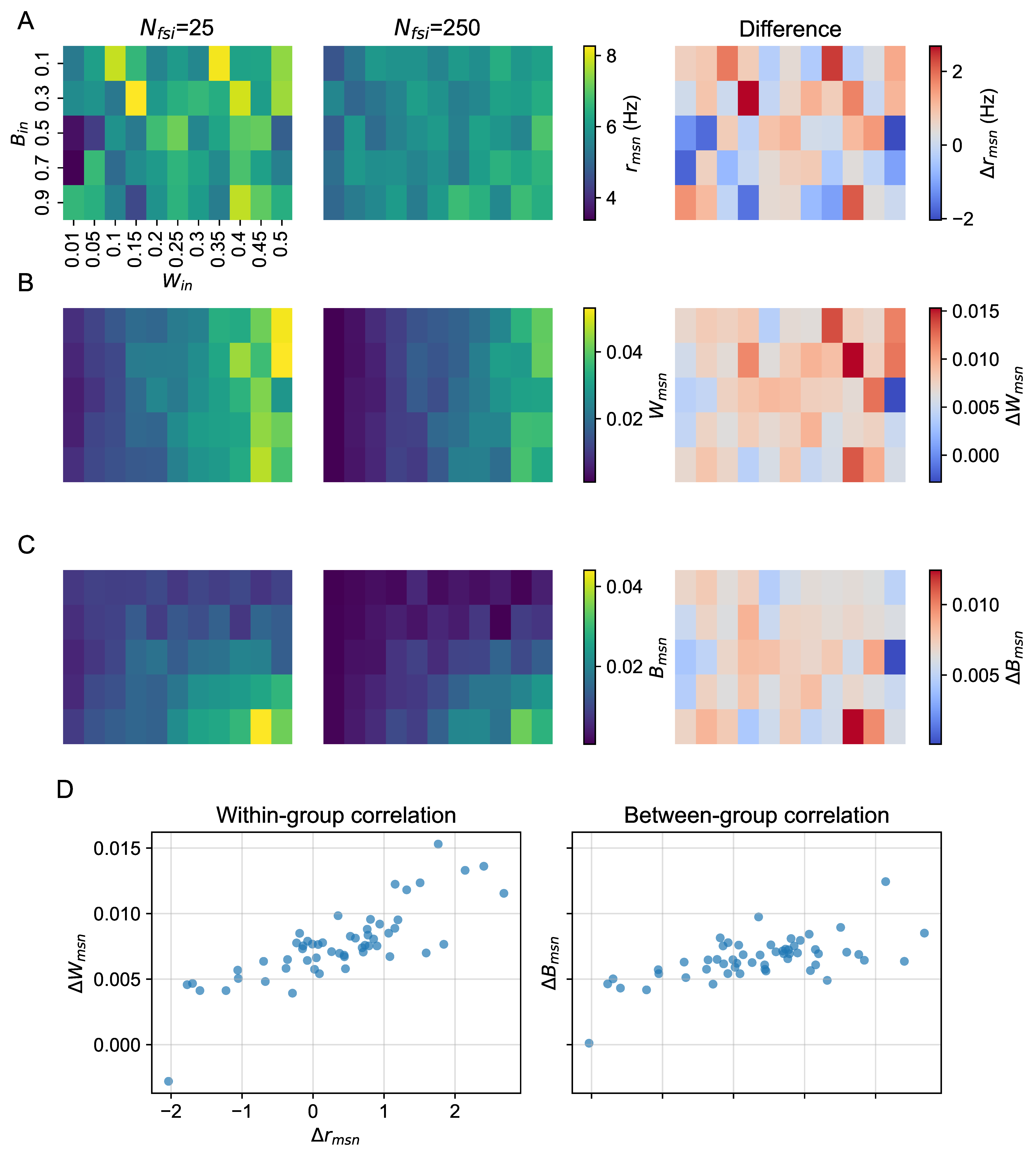

Supplement: S5 Fig — (TIFF) [file pcbi.1014099.s005.tiff]

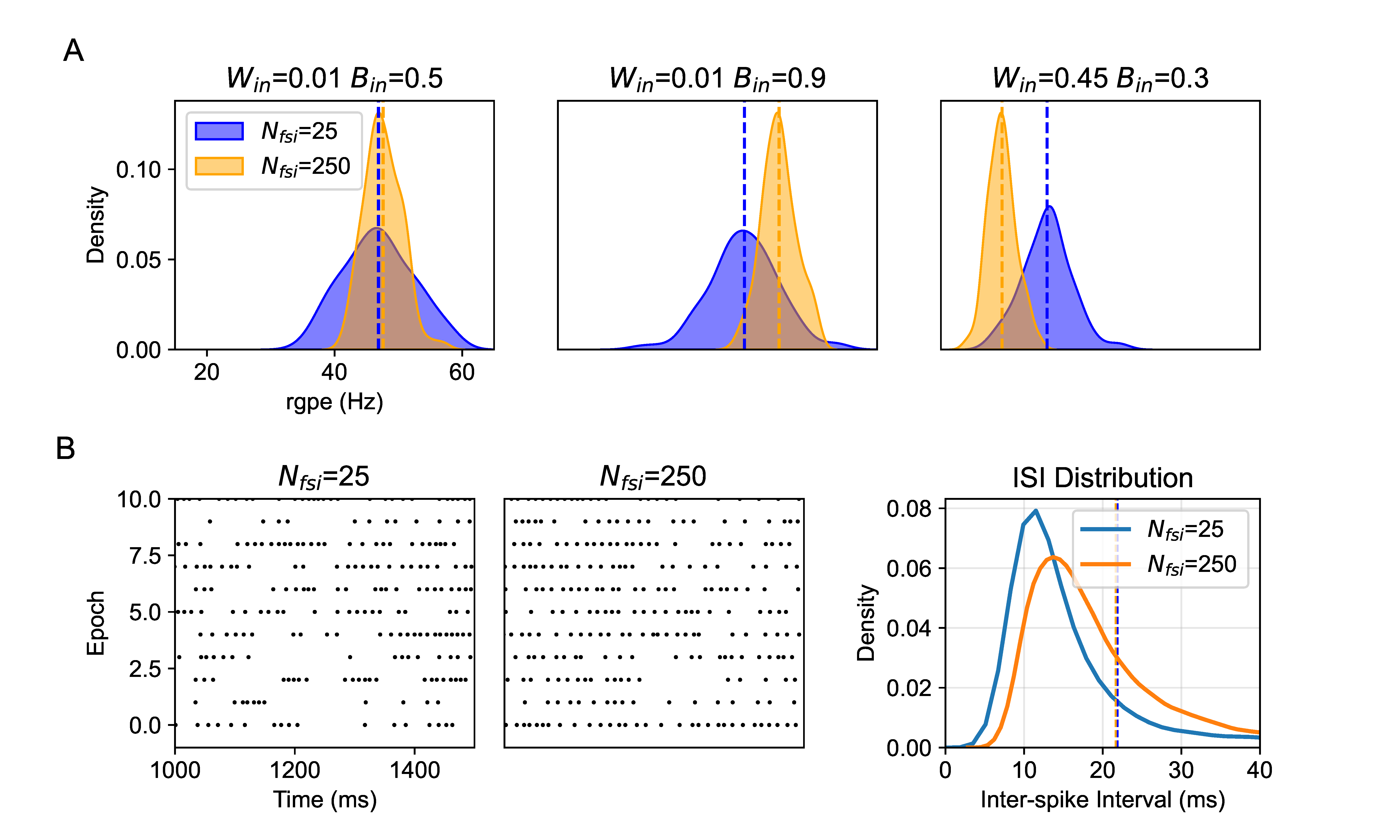

Supplement: S6 Fig — (TIFF) [file pcbi.1014099.s006.tiff]

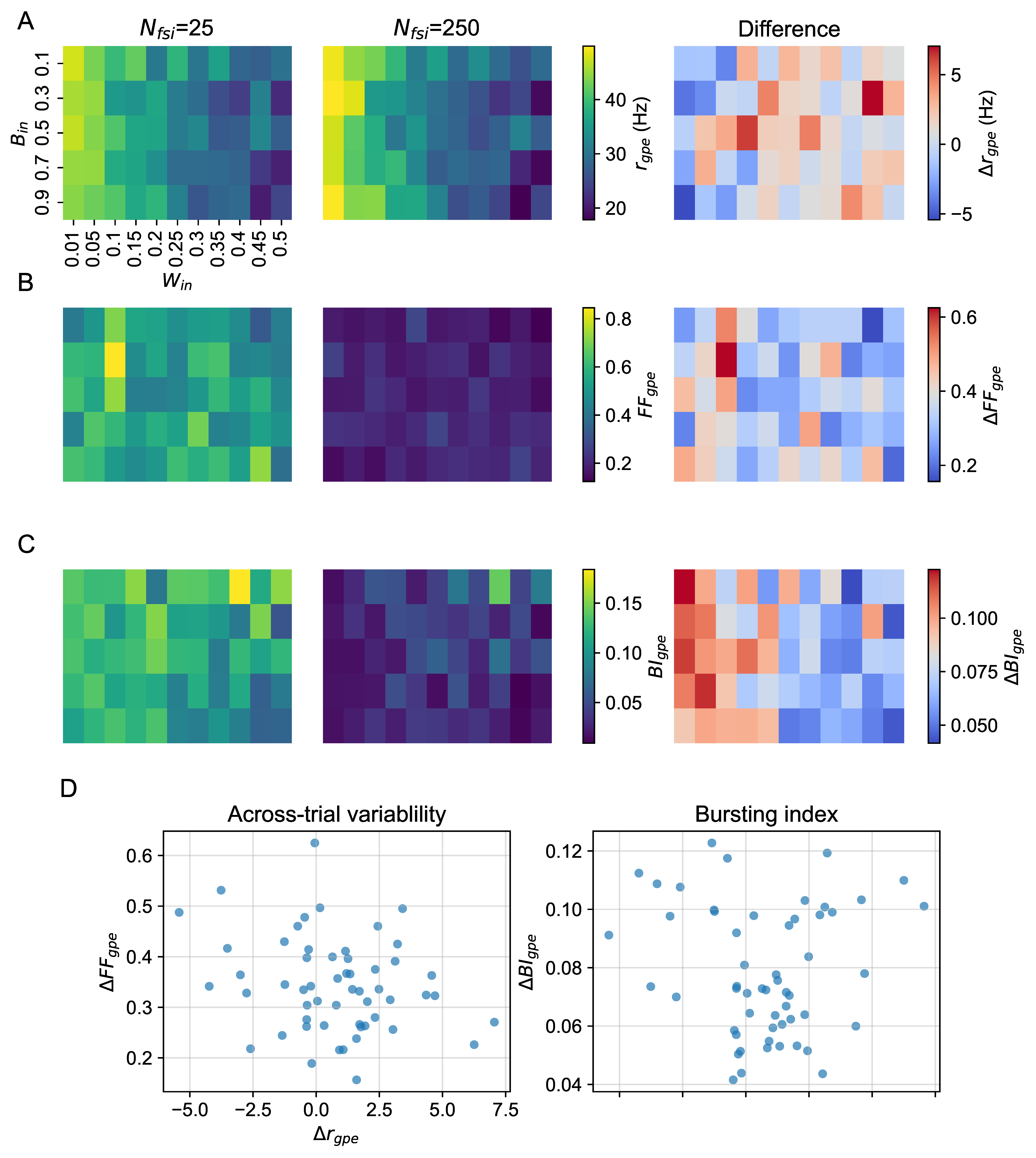

Supplement: S7 Fig — (TIFF) [file pcbi.1014099.s007.tiff]

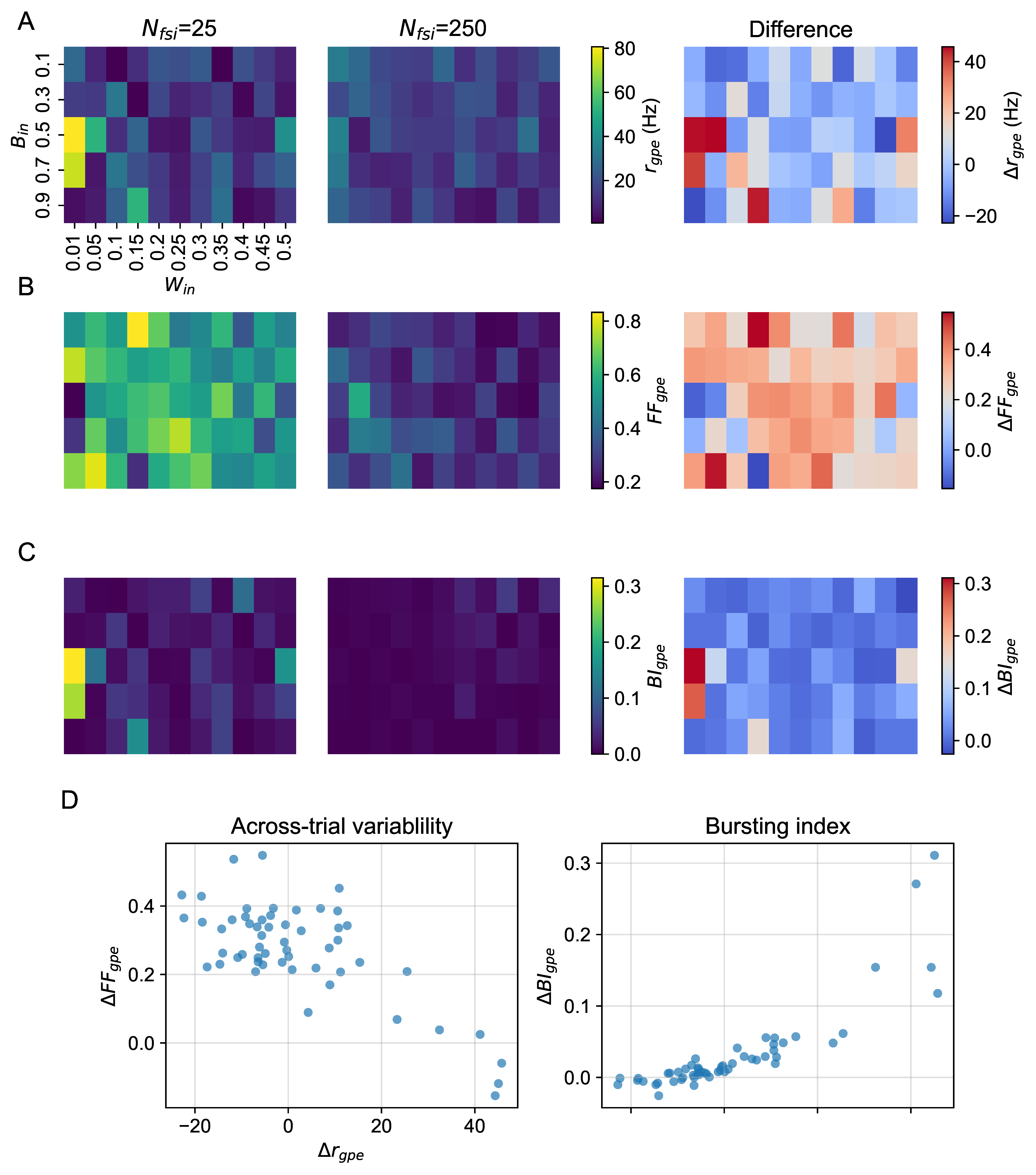

Supplement: S8 Fig — (TIFF) [file pcbi.1014099.s008.tiff]
